# Gd^3+^ and Bi^3+^ co-substituted cubic zirconia; (Zr_1−*x*−*y*_Gd_*x*_Bi_*y*_O_2−*δ*_): a novel high κ relaxor dielectric and superior oxide-ion conductor

**DOI:** 10.1039/d2ra01867e

**Published:** 2022-05-13

**Authors:** Akanksha Yadav, Rajiv Prakash, Preetam Singh

**Affiliations:** Department of Ceramic Engineering, Indian Institute of Technology (Banaras Hindu University) Varanasi Varanasi 221005 India preetamsingh.cer@itbhu.ac.in preetamchem@gmail.com +91-9473720659; School of Materials Science and Technology, Indian Institute of Technology (Banaras Hindu University) Varanasi Varanasi 221005 India

## Abstract

Solid oxide fuel cells (SOFCs) offer several advantages over lower temperature polymeric membrane fuels cells (PMFCs) due to their multiple fuel flexibility and requirement of low purity hydrogen. In order to decrease the operating temperature of SOFCs and to overcome the high operating cost and materials degradation challenges, the Cubic phase of ZrO_2_ was stabilized with simultaneous substitution of Bi and Gd and the effect of co-doping on the oxide-ion conductivity of Zr_1−*x*−*y*_Bi_*x*_Gd_*y*_O_2−*δ*_ was studied to develop a superior electrolyte separator for SOFCs. Up to 30% Gd and 20% Bi were simultaneously substituted in the cubic ZrO_2_ lattice (Zr_1−*x*−*y*_Gd_*x*_Bi_*y*_O_2−*δ*_, *x* + *y* ≤ 0.4, *x* ≤ 0.3 and *y* ≤ 0.2) by employing a solution combustion method followed by multiple calcinations at 900 °C. Phase purity and composition of the material is confirmed by powder XRD and EDX measurements. The formation of an oxygen vacant Gd/Bi co-doped cubic zirconia lattice was also confirmed by Raman spectroscopy study. With the incorporation of Bi^3+^ and Gd^3+^ ions, the cubic Zr_1−*x*−*y*_Bi_*x*_Gd_*y*_O_2−*δ*_ phase showed relaxor type high κ dielectric behaviour (*ε*′ = 9725 at 600 °C at applied frequency 20 kHz for Zr_0.6_Bi_0.2_Gd_0.2_O_1.8_) with *T*_m_ approaching 600 °C. The high polarizability of the Bi^3+^ ion coupled with synergistic interaction of Bi and Gd in the host ZrO_2_ lattice seems to create the more labile oxide ion vacancies that enable superior oxide-ion transport resulting in high oxide ion conductivity (*σ*_o_ > 10^−2^ S cm^−1^, *T* > 500 °C for Zr_0.6_Bi_0.2_Gd_0.2_O_1.8_) at relatively lower temperatures.

## Abstract 2

The high polarizability of the Bi^3+^ ion coupled with synergistic interaction of Bi and Gd in the host ZrO_2_ lattice seems to create the more labile oxide ion vacancies that enable high oxide ion conductivity at lower temperatures.

## Graphical abstract

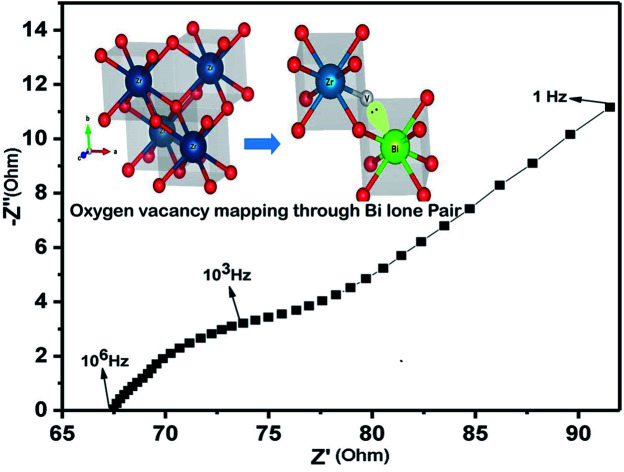

## Introduction

Since after the discovery of superior oxide-ion conductivity in Mg doped perovskite structure Na_0.5_Bi_0.5_TiO_3_,^1^ a well known piezoelectric material that possesses high leakage conductivity that makes the material unsuitable for piezo- and ferroelectric applications, newer interest is open to developing superior oxide-ion conducting materials through controlling the nature of dielectricity of the materials. The fast oxygen ion diffusion of Na_0.5_Bi_0.5_TiO_3_ (NBT) is attributed to the high polarizability of Bi^3+^ and is mediated by oxygen vacancies^1^ that can be introduced either by changing the NBT compositions through Bi deficiency or by Mg doping.^1,2^ Dielectric leakage or relaxor-like characteristics of ferroelectrics or high κ dielectric materials reveal as a strong temperature and frequency dependence in the maximum of both real and imaginary parts of the dielectric permittivity. However, relaxors not only show particular and intriguing behaviours in the dielectric response, but also show promising activity in fast-ion conduction to be applied as oxide-ion conductors for application in solid oxide fuel cells (SOFCs), oxygen separation membranes, oxygen sensors and oxygen pumps.^3–9^

Intermediate temperature solid oxide fuel cells (IT-SOFCs) have gained recent attention due to their potential long-term durability, shorter start-up times and economic competitiveness for a wide range of applications, such as small-scale portable devices, automotive auxiliary power units and large distributed power generation systems.^10–15^ However, significant increases in power losses factors especially ohmic and activation losses, due to relatively high temperature (*T* > 800 °C) operations reduce the cell performance.^16–19^ The ohmic and activation losses are primarily related to oxide-ion transport through the electrolyte and the sluggish reaction kinetics on the electrode surfaces. These losses can be reduced by using the electrolyte materials with high ionic conductivity at low temperatures, reducing electrolyte thickness, increasing reactant concentration, and a number of potential reaction sites, and decreasing the activation barrier. In the past few decades, significant research has been done in the development of perovskite and fluorite based oxide ionic conductors, *e.g.*, LaGaO_3_ based (Sr and Mg doped) perovskites,^20–23^ rare earth doped ceria based materials,^6,9,24–26^ Na_0.5_Bi_0.5_Ti_1−*x*_Mg_*x*_O_3−*δ*_,^1–3,27,28^ δ-Bi_2_O_3_,^4,29–32^ KTa_0.4_Ti_0.3_Ge_0.3_O_2.7_.^5^

Ceria-based materials, especially rare earth-doped ceria (GDC and SDC), have been considered strong candidates for IT-SOFCs electrolytes due to their high ionic conductivity in intermediate temperature range. But their performance suffers/degrades due to electronic conduction resulting through partial reduction of Ce^4+^ into Ce^3+^ at low oxygen partial pressures.^33,34^ This chemical instability of ceria restricts the application of the electrolyte resulting the issue of the stability of the cell. Further, high cost of gallium and formation of inactive secondary phases during the preparation of LaGaO_3_-based electrolyte is a serious concern that hamper the applicability of the material as an oxide-ion electrolyte in SOFCs. That is why more attention is given on the fabrication of thin electrolytes supported SOFCs relying on yttria-stabilized zirconia (YSZ) that has been widely used as an electrolyte material at high temperatures rather than the ceria-based electrolyte.^35–40^

In ZrO_2_-based materials, combination of high dielectric permittivity and thermal stability with low leakage current due to a reasonably high barrier height that limits electron tunnelling, counts it to further research as oxide ion conductor for SOFCs application.^36–38^ Also, Bi-based based oxide ion conductors demonstrate the remarkable ionic conductivity due to high-concentration of intrinsic oxygen vacancies and high polarizability of Bi^3+^ with 6s^2^ lone pair electrons.^39^ A zirconia-based electrolyte (YSZ) is considered the most effective candidate as a solid electrolyte for electrochemical cells working either in open-circuit mode (oxygen sensor) or in a power application (oxygen pump and solid-oxide fuel cell) due to its robustness.^35–40^ However; it seems to be an arduous task to achieve IT-SOFCs at a commercial scale using YSZ based electrolytes due to its relatively low oxide-ion conductivity at intermediate temperatures.

Recent studies demonstrate that the relaxor nature of high κ dielectricity and higher polarizability of Bi^3+^ ion seems to play a directive role in providing superior oxide-ion transport throughout the lattice at temperature close to dielectric relaxation temperatures.^1,4,47^ ZrO_6_ octahedra was a feudal point in developing superior high κ dielectric/ferroelectric materials especially in PZT based perovskite structures. Relaxor type of high dielectric materials were also reported for doped cubic zirconia phases.^41–44^ We have envisaged that high polarizability of Bi^3+^ ion couple with high κ dielectric relaxation (high dielectric leakage) can generate superior oxide-ion conduction near *T*_m_ (the temperature of the maximum dielectric permittivity). To realize the concept, we attempted the suitable dopping of Bi^3+^ and Gd^3+^ ions into ZrO_2_ lattice to stabilize cubic phase of zirconia and found that the synergistic interaction by introducing a secondary substituent (Gd^3+^ ions) enhances the oxide-ion vacancy transport within the percolation limit of ion transport inside the host structure at lower temperatures. In cubic ZrO_2_, the theoretical ratio of the ionic radius of the cation to anion (O^2−^) for fully packed FCC lattice is 0.73 at room temperature, but the ratio is 0.59 for tetragonal phase of ZrO_2_ stabilized at room temperature.^45^ Hence, doping of other elements with larger ionic radius than Zr at Zr site is an efficient way to stabilize the high-temperature cubic phase at room temperature by the formation of solid solutions. Our study show that the co-doping of Bi^3+^ and Gd^3+^ ions (ionic radii in 8 coordinations, Bi^3+^ = 1.17 Å and Gd^3+^ = 1.053 Å)^46^ in ZrO_2_ lattice resulted the formation of Zr–Bi–Gd–O solid solution in cubic fluorite structure at room temperature and also it resulted the superior oxide-ion transport (oxide ion conductivity ∼ 10^−2^ S cm^−1^ above 500 °C) at lower temperatures. Material also showed relaxor type dielectric nature of solid solution coupled with synergistic interaction of Gd and Bi in solid solution Zr_1−*x*−*y*_Bi_*x*_Gd_*y*_O_2−*δ*_. Here, we present the synthesis, characterization, permittivity and oxide-ion conductivity studies of Bi^3+^ and Gd^3+^ substituted cubic zirconia in this manuscript.

## Experimental

### Material's synthesis and characterization

Zr_1−*x*−*y*_Bi_*x*_Gd_*y*_O_2−*δ*_ samples were synthesized by employing solution combustion method by dissolving stoichiometric amount of ZrO(NO_3_)·*x*H_2_O, Bi_2_O_3_ and Gd_2_O_3_ in 100 ml of 40% nitric acid solution with continuous stirring at 90 °C for 4–5 hours. Further for auto-combustion, glycine was used as the fuel and was added in a molar ratio of 1.5 : 1 to total moles of metal ions present in the solution. The temperature of the hot plate-magnetic stirrer was increased to 250 °C for combustion to start. Reaction ends up with vigorous combustion after the evaporation of water at gelation point due to constant heating. The material left behind after combustion was collected, and multiple calcinations were carried out at 900 °C for 12 hours to get single-phase materials. For conductivity measurement, the powder was made into pallets of 10 mm diameter and ∼0.2 cm thickness by pressing it to ∼8 ton weight on a hydraulic press. These pallets were fired at 1000 °C for 10 hours for densification. Density of the pellet was measured by using Archimedes method and it was found to be ∼97% of the apparent density obtained from geometrical analysis.

The phase formation study was carried out through Rigaku Miniflex desktop X-ray diffractometer (XRD) with Cu Kα radiation (*λ* = 1.54 Å) in the range 2*θ* ∼ 10–90° with a step size of 0.02°. The structures were refined by the Rietveld refinement method using the FULLPROF suite software package and cubic fluorite ZrO_2_ (space group: *Fm*3̄*m*) as model structure. The microstructures of the sintered samples were investigated by using scanning electron microscopy (EVO – scanning electron microscope MA15/18). The average grain size was calculated using the linear intercept method. The composition of the compounds was examined by energy dispersive X-ray (EDX) spectroscopy with a probe attached to the SEM instrument. Raman spectroscopy of powdered sample was carried out by using STR-300 micro-Raman spectrometer with a laser excitation wavelength of 532 nm and step size of 1.9 cm^−1^.

Pt paste was used as a blocking electrode for conductivity measurements. For this purpose, the sintered pellets were coated with platinum paste and cured at 800 °C for 30 minutes. The conductivity measurements were performed using Autolab potentiostat as a function of frequency from 1 MHz to 1 Hz at different temperatures varying from 100 °C to 650 °C. All measurements were taken during the cooling cycle from 650 °C to 100 °C.

## Result and discussion

**Fig. 1:**
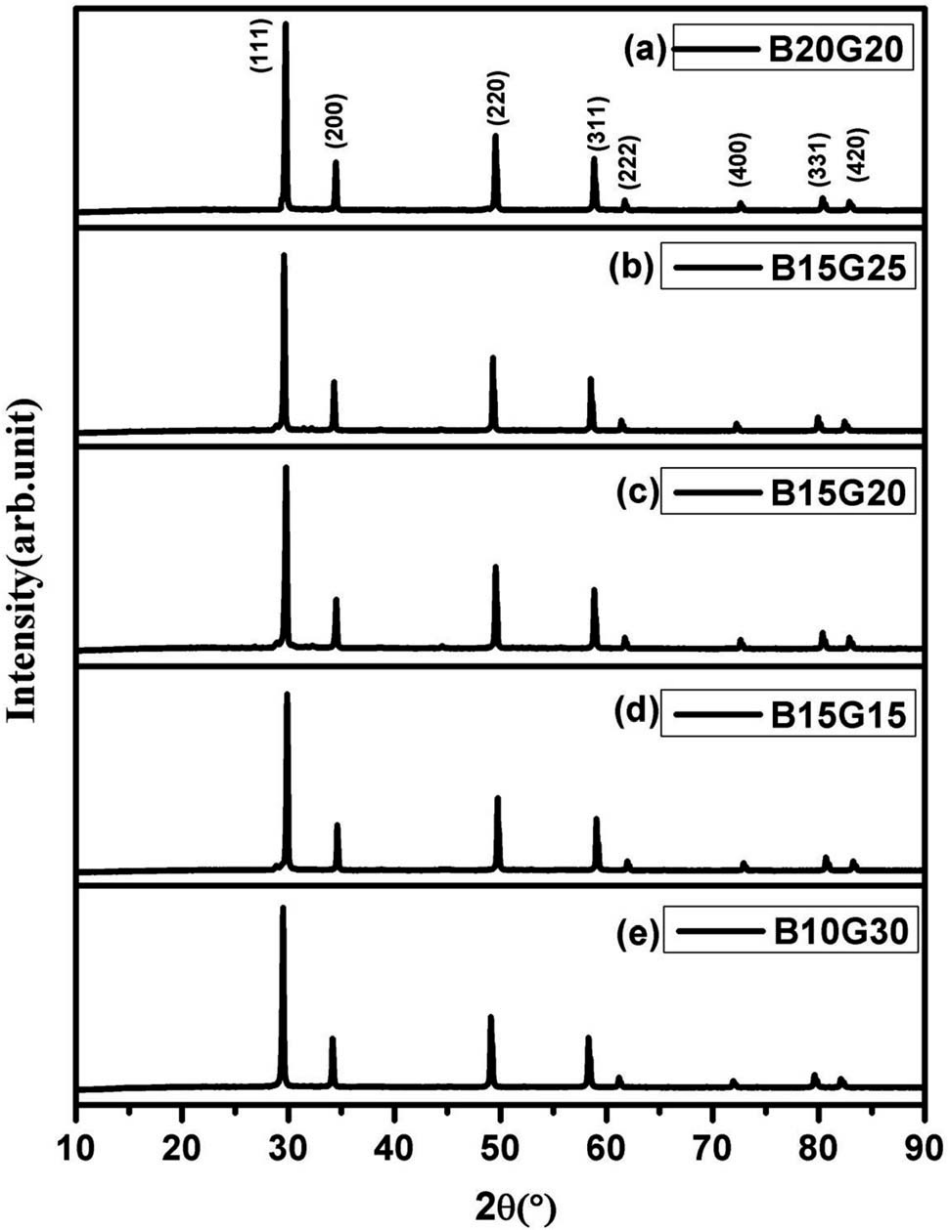
Powder XRD pattern (a) Zr_0.6_Bi_0.2_Gd_0.2_O_1.8_, (b) Zr_0.6_Bi_0.15_Gd_0.25_O_1.8_, (c) Zr_0.65_Bi_0.15_Gd_0.2_O_1.825_, (d) Zr_0.7_Bi_0.15_Gd_0.15_O_1.85_, and (e) Zr_0.6_Bi_0.10_Gd_0.30_O_1.8_.

**Fig. 2:**
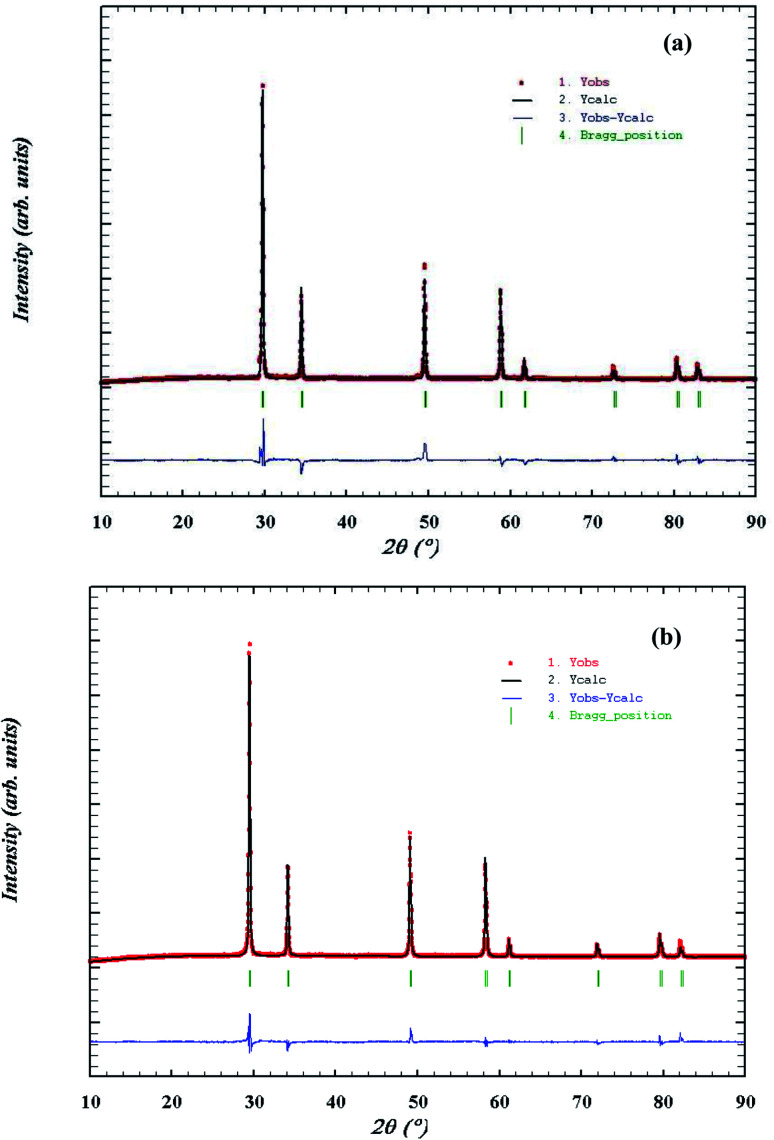
Rietveld refined powder XRD profile of (a) Zr_0.6_Gd_0.2_Bi_0.2_O_1.8_, (b) Zr_0.6_Gd_0.30_Bi_0.10_O_1.8_.

As Gd^3+^ ions can stabilized the ZrO_2_ in cubic fluorite structure,^37^ role or promoting effect of Bi^3+^ ion were utilized to develop superior oxide-ion conductors. Several compositions of Zr_1−*x*−*y*_Gd_*x*_Bi_*y*_O_2−*δ*_ in cubic fluorite structure were synthesized and few important data were presented in the manuscript. We have found that at max, total 40% ions can be substituted at Zr site to make single phase cubic fluorite material using Gd^3+^ and Bi^3+^ as simultaneous substituent. Thus, up to 20% of Bi and 30% Gd was co-substituted in ZrO_2_ lattice (Zr_1−*x*−*y*_Gd_*x*_Bi_*y*_O_2−*δ*_, *x* + *y* ≤ 0.4, *x* ≤ 0.3 and *y* ≤ 0.2) in different combinations and several solid solutions were synthesized in cubic fluorite structure. The synthesized Bi^3+^ and Gd^3+^ substituted ZrO_2_ powder was in off-white in colour. The crystal structure and phase purity of the material was analyzed by powder XRD study. Powder XRD pattern of Zr_0.6_Bi_0.2_Gd_0.2_O_2−*δ*_ (B20G20), Zr_0.6_Bi_0.15_Gd_0.25_O_2−*δ*_ (B15G25), Zr_0.65_Bi_0.15_Gd_0.20_O_2−*δ*_ (B15G20), Zr_0.7_Bi_0.15_Gd_0.15_O_2−*δ*_ (B15G15), and Zr_0.6_Bi_0.10_Gd_0.30_O_2−*δ*_ (B10G30), are shown Fig. 1(a–e) respectively. All the peaks were identified to cubic fluorite yttria stabilized zirconia (YSZ) structure (JCPDS no. 98-001-9128). No impurity peaks were identified for Gd_2_O_3_, Bi_2_O_3_ or any other phases of pure ZrO_2_. Thus single phase materials were synthesized using solution combustion route with multiple calcinations at 900 °C for 12 hours. Crystal structures of Bi and Gd substituted ZrO_2_ were refined using the Rietveld method. Fig. 2 shows the representative Rietveld refined XRD profile for (a) Zr_0.6_Bi_0.2_Gd_0.2_O_1.8_ and (b) Zr_0.6_Bi_0.1_Gd_0.3_O_1.8_. Fitted profile matched well with the observed XRD pattern. The structural parameters obtained from Rietveld refinement of powder XRD pattern is given in Table 1. Due to substitution of larger Bi^3+^ and Gd^3+^ cations on Zr site in ZrO_2_ lattice, there was increase in the lattice parameter of the materials with increase in concentration of dopants.

**Table 1:** Structural parameter of Bi and Gd doped ZrO_2_

Compound	Lattice parameter (Å) (*a* = *b* = *c*)	*χ* ^2^	*R*_f_	*R*_Bragg_
ZrO_2_	5.13 (ref. 45)	—	—	—
Zr_0.6_Bi_0.2_Gd_0.2_O_1.8_	5.2444	2.86	3.51	5.18
Zr_0.6_Bi_0.15_Gd_0.25_O_1.8_	5.2362	3.07	4.96	7.49
Zr_0.65_Bi_0.15_Gd_0.2_O_1.825_	5.2298	3.43	6.29	8.71
Zr_0.7_Bi_0.15_Gd_0.15_O_1.85_	5.2392	4.12	7.23	9.54
Zr_0.6_Bi_0.10_Gd_0.30_O_1.8_	5.2021	4.94	9.54	12.41

**Fig. 3:**
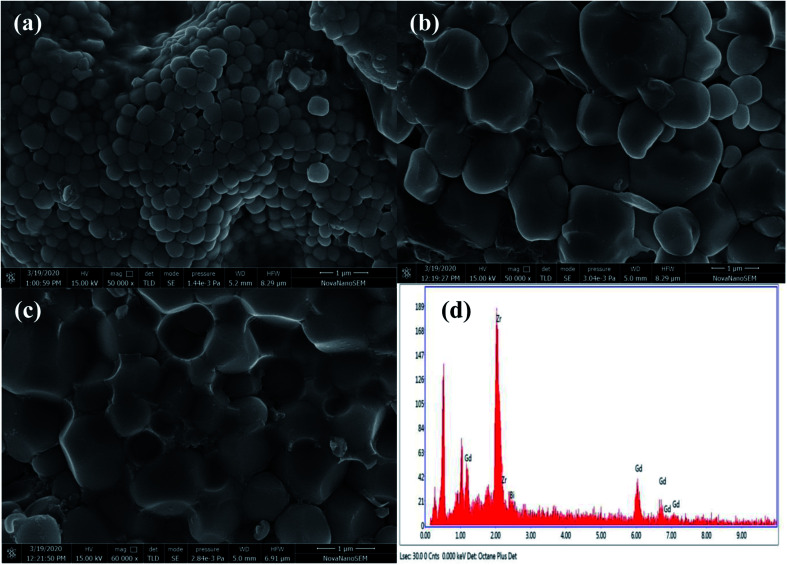
SEM image Zr_0.6_Bi_0.2_Gd_0.2_O_1.8_ (a) powder (b) front view of the pellet (c) cross section of the pallets and (d) EDX image of the pallet.

SEM micrographs of Zr_0.6_Bi_0.2_Gd_0.2_O_1.8_ (powder, top view and cross section of the pellet used for conductivity measurements) are shown in Fig. 3(a–c). The SEM study shows that the powders are made of with interconnected grains in size of 4–10 μm. Fig. 3(b and c) show the top and cross section images of the pellet. Crystal growth during sintering resulted microstructure is having nearly no or very low porosity and the grains are thoroughly interconnected (good contact with each other). Further, no color contrasts was observed in the SEM images representing the uniform distribution of elements in the grains of the materials. The EDX study of the Zr_0.6_Gd_0.2_Bi_0.2_O_2−*δ*_ sample (micrograph shown in Fig. 3(d)) confirms that the elements Zr : Gd : Bi were present in the ratios of 0.589 : 0.195 : 216 that is very much close to the elemental ratios used for the synthesis.

Thus XRD study and SEM study coupled with EDX study confirms that Bi^3+^ and Gd^3+^ ions are substituted at Zr^4+^ sites in stabilized cubic ZrO_2_ lattice. Considering the Schottky defect formation due to substitution of Bi^3+^ and Gd^3+^ at Zr^4+^ sites that will create oxygen vacancy generation in the lattice and the oxygen defect formation equation using Kröger–Vink notation can be represented as:1
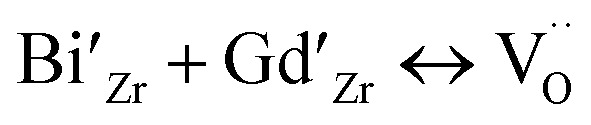


**Fig. 4:**
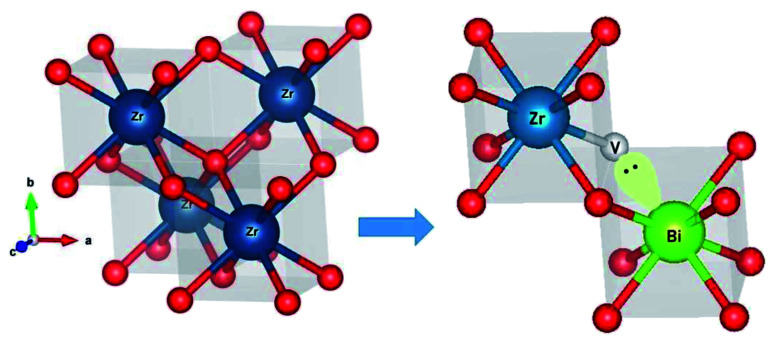
Oxygen vacancy mapping through Bi lone pair in cubic fluorite structure.

Further the lone pair of Bi^3+^ ions can stabilize the oxide-ion vacant fluorite structure as represented in Fig. 4. The lone pair of Bi^3+^ ion are known to implant higher vacancy mobility as it was witnesses in the case of Bi based oxide-ion conductors.^1,4,27–31,47^

**Fig. 5:**
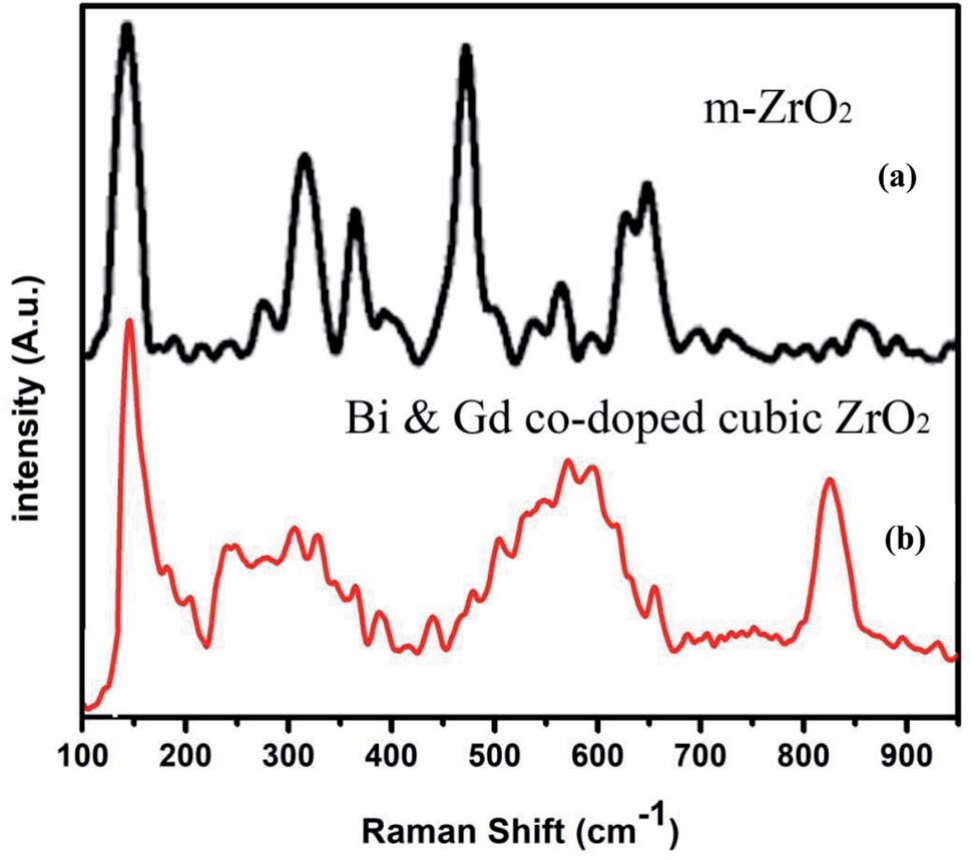
Raman spectra of (a) monoclinic ZrO_2_ and (b) Gd–Bi co-doped cubic zirconia (Zr_0.6_Bi_0.2_Gd_0.2_O_1.8_).

Fig. 5 presents the Raman spectra of (a) undoped monoclinic zirconia and (b) Gd/Bi co-doped cubic zirconia (Zr_0.6_Bi_0.2_Gd_0.2_O_1.8_). The Raman spectrum for cubic ZrO_2_ is characterized by a narrow band at 145 cm^−1^ and broad bands centered around 250, 305, 440, and ∼601 cm^−1^. The stabilized ZrO_2_ sample in this study clearly showed the broad peak between 500 to 650 cm^−1^ that is related to the disordered oxygen sub-lattice along with mass-related disorder indicating of a large disorder in the cationic cage upon Gd and Bi ion substitution in cubic ZrO_2_ lattice whereas monoclinic ZrO_2_ exhibits several well defined sharp bands because of the symmetry reduction.^48^ Since the cations are much heavier than the oxygen atoms, they are the major contributors to the vibrations associated with the acoustic branches indicating a periodic arrangement of the vacancies in stabilized Gd/Bi co-doped cubic-zirconia the lattice.^49–51^ A careful examination of the Raman spectra also shows weak bands around 620, 660, and 815 cm^−1^ that could be associated with the rearrangement of the anionic sub-lattice, *i.e.* oxygen ions and vacancies containing Bi cage in stabilized Bi/Gd codoped cubic zirconia.^52^ Further observed bands around 535 nm and 790 nm can be assigned to Raman vibrations of Gd containing sub lattice of stabilized Bi/Gd codoped cubic zirconia.^53^ Thus the Raman spectroscopy study clearly reveal the formation of oxygen vacant Gd/Bi co-doped cubic zirconia lattice.

**Fig. 6:**
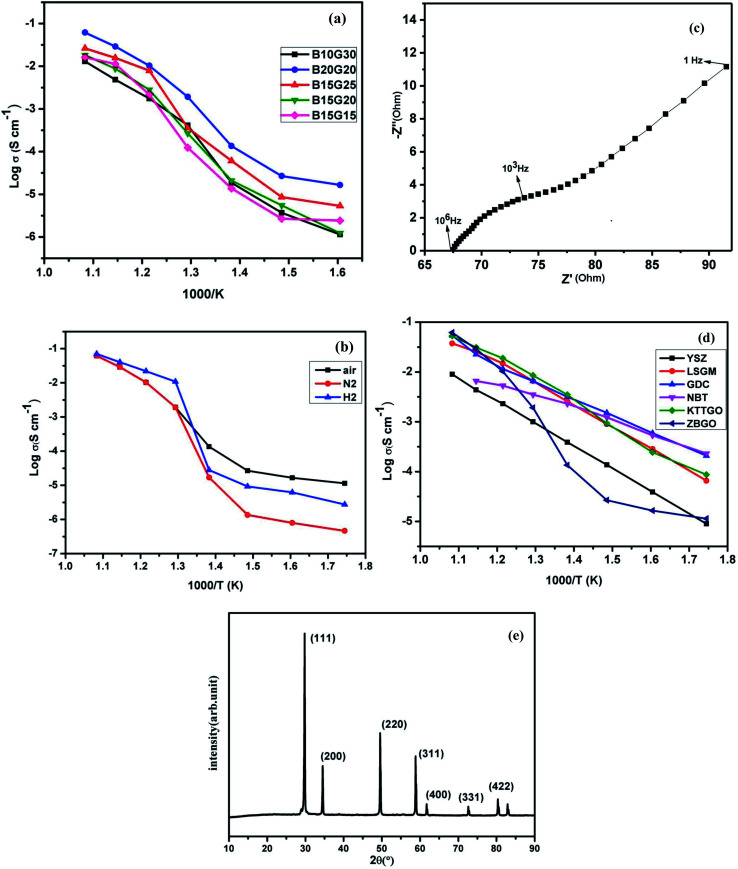
Arrhenius plot of (a) various compositions, (b) Zr_0.6_Bi_0.2_Gd_0.2_O_1.8_ (ZBGO) in different medium, (c) Cole–Cole plot of Zr_0.6_Bi_0.2_Gd_0.2_O_1.8_ at 500 °C, (d) comparison of existing intermediate temperature oxide ion electrolytes in air (data taken from ref. 1) and (e) powder XRD pattern of Zr_0.6_Bi_0.2_Gd_0.2_O_1.8_ heated in hydrogen atmosphere up 800 °C during impedance study.

To see the effect of Gd and Bi substitution in the cubic fluorite ZrO_2_ structure, impedance spectroscopy was utilized to study the oxide-ion vacancy conduction process and dielectric constant of the materials at various temperatures in different environments. Fig. 6(a) show the conductivity plot for different composition of Bi and Gd substituted ZrO_2_*i.e.* (i) Zr_0.6_Bi_0.2_Gd_0.2_O_1.8_ (B20G20), (ii) Zr_0.6_Bi_0.15_Gd_0.25_O_1.8_ (B15G25), (iii) Zr_0.65_Bi_0.15_Gd_0.2_O_1.825_ (B15G20), (iv) Zr_0.7_Bi_0.15_Gd_0.15_O_1.85_ (B15G15), and (v) Zr_0.6_Bi_0.10_Gd_0.30_O_1.8_ (B10G30). The total electrical conductivity was found continuously increasing with increasing temperature for all the compositions. The best electrical conductivity of this series was observed for the composition Zr_0.6_Bi_0.2_Gd_0.2_O_1.8_. At 550 °C, the measured conductivity was ∼10^−2^ S cm^−1^ for Zr_0.6_Bi_0.2_Gd_0.2_O_1.8_, which is better than that of Zr_0.92_Y_0.08_O_2_ (YSZ) at 670 °C at and of La_0.8_Sr_0.2_Ga_0.83_Mg_0.17_O_3_ (LSGM) at 600 °C. Here, a careful study was made on development to superior ZrO_2_ based electrolyte. As Gd can stabilized the ZrO_2_ in cubic fluorite structure, role or promoting effect of Bi^3+^ ion were utilized to develop superior oxide-ion conductors. Systematically, we carried out ionic conductivity study of Gd stabilized ZrO_2_ for 15%, 20%, 25%, 30% Gd^3+^ ions doping in ZrO_2_ and ionic conductivity data of these materials are provided in Table 2. It was found that 20 and 25% Gd stabilized cubic ZrO_2_ showed almost similar conductivities. Further to improve the conductivity of Gd stabilized cubic ZrO_2_, Gd^3+^ and Bi^3+^ion co-substituted cubic zirconia was synthesized. We have found that at max, total 40% ions can be substituted at Zr site to make single phase cubic fluorite material. In first attempt additional 15% Bi^3+^ co-doping was attempted along with Gd^3+^ ions. However, in case of 30% Gd stabilized zirconia, only 10% additional Bi^3+^ can be doped in single phase. Among them, we Zr_0.65_Gd_0.20_Bi_0.15_O_2−*δ*_ showed highest conductivity. Further we extended Bi^3+^ ion substitution in Gd stabilized cubic zirconia and found that highest conductivity can be achieved with Zr_0.6_Gd_0.2_Bi_0.2_O_2−*δ*_ sample. Thus this study can confirm that maximum 40% substitution in ZrO_2_ lattice can be achieved using Gd^3+^ and Bi^3+^ ions together, and the highest conductivity was achieved for cubic fluorite Zr_0.6_Gd_0.2_Bi_0.2_O_2−*δ*_ sample. The data for oxide-ion conductivity of different samples of Zr_1−*x*−*y*_Bi_*x*_Gd_*y*_O_2−(*x*+*y*)/2_ at different temperature along with the data of other competitive oxide-ion electrolyte in the same temperature range is given in Table 2. As shown in Table 2, highest conductivity (1.1 × 10^−2^ S cm^−1^ at 550 °C) was observed for Zr_0.6_Gd_0.2_Bi_0.2_O_2−*δ*_. As evident from the study, the conductivity of the materials was increased with increasing Gd content in the cubic fluorite ZrO_2_ lattice. At initiation of co-doping, the promoting effect of Bi is clearly visible on co-doping of Bi along with Gd in ZrO_2_ lattice and we found that the maximum conductivity was observed for Zr_0.6_Gd_0.2_Bi_0.2_O_2−*δ*_ sample as maximum co-doping or simultaneous substitution of Gd and Bi in cubic fluorite ZrO_2_ lattice is limited to 40%.

**Table 2:** O^2−^ conductivity (*σ*) of Zr_1−*x*−*y*_Bi_*x*_Gd_*y*_O_2−*δ*_ at different temperatures

Conductivity (S cm^−1^)				
Compound	650 °C	600 °C	550 °C	500 °C
Zr_0.85_Gd_0.15_O_1.925_	1.0 × 10^−2^	3.5 × 10^−3^	1.2 × 10^−3^	3.2 × 10^−4^
Zr_0.80_Gd_0.20_O_1.9_	1.4 × 10^−2^	4.4 × 10^−3^	1.9 × 10^−3^	4.3 × 10^−4^
Zr_0.75_Gd_0.25_O_1.875_	1.1 × 10^−2^	4.9 × 10^−3^	1.6 × 10^−3^	4.8 × 10^−4^
Zr_0.70_Gd_0.30_O_1.85_	1.0 × 10^−3^	3.8 × 10^−3^	2.1 × 10^−3^	2.2 × 10^−4^
**Zr**_**0.6**_ **Bi**_**0.2**_ **Gd**_**0.2**_ **O**_**1.8**_	6.2 × 10^−2^	3.2 × 10^−2^	1.1 × 10^−2^	1.9 × 10^−3^
Zr_0.6_Bi_0.15_Gd_0.25_O_1.8_	2.6 × 10^−2^	1.6 × 10^−2^	7.9 × 10^−3^	3.6 × 10^−4^
Zr_0.65_Bi_0.15_Gd_0.2_O_1.825_	1.8 × 10^−2^	8.8 × 10^−3^	2.8 × 10^−3^	2.7 × 10^−4^
Zr_0.6_Bi_0.15_Gd_0.15_O_1.85_	1.6 × 10^−2^	1.2 × 10^−2^	2.3 × 10^−3^	1.2 × 10^−4^
Zr_0.7_Bi_0.10_Gd_0.30_O_1.8_	1.2 × 10^−2^	4.8 × 10^−3^	1.8 × 10^−3^	4.2 × 10^−4^
KTa_0.4_Ti_0.3_Ge_0.3_O_2.7_ (ref. 37)	5.2 × 10^−2^	3.1 × 10^−2^	9.8 × 10^−3^	8.5 × 10^−3^
Na_0.5_Bi_0.49_Ti_0.98_Mg_0.02_O_2.965_ (ref. 29)		6.6 × 10^−3^	5.4 × 10^−3^	3.5 × 10^−3^
Zr_0.92_Y_0.08_O_1.96_ (ref. 29)		4.4 × 10^−3^	2.3 × 10^−3^	1 × 10^−3^
Ce_0.9_Gd_0.1_O_1.95_ (ref. 29)		2.3 × 10^−2^	1.2 × 10^−2^	6.5 × 10^−3^
La_0.9_Sr_0.1_Ga_0.9_Mg_0.1_O_2.9_ (ref. 29)		2.5 × 10^−2^	1.5 × 10^−2^	6.5 × 10^−3^

Impedance study of Zr_0.6_Bi_0.2_Gd_0.2_O_1.8_ was also carried out at different temperatures in dry hydrogen (UHP H_2_) and dry nitrogen (UHP N_2_) environment (Fig. 6(b)) also to see the effect of absorbed moisture, and impurities present in the air on the surface or at oxide-ion vacancy sites of the sample and also the stability of the material in reducing environment in presence of hydrogen. Below 500 °C, the total conductivity of Zr_0.6_Bi_0.2_Gd_0.2_O_1.8_ was found little lower in hydrogen and nitrogen atmosphere compared to air. The cubic fluorite phase of ZrO_2_, YSZ is predominantly a total oxide-ion conductor. Below 500 °C, Gd and Bi doped ZrO_2_ sample in air atmosphere may have little bit associate protonic conduction contribution due to presence of existing moist into the air. As moisture present in air can result absorption of moisture on the surface of the sample at low temperature contributing to additional conductivity at those temperatures. As we have not found any increase in total conductivity in hydrogen atmosphere even at higher temperatures, this suggests the stability of Bi^3+^ ion in ZrO_2_ lattice that does not allow the reduction Bi^3+^ ions in hydrogen media. The Cole–Cole plot at 500 °C in air atmosphere for Zr_0.6_Bi_0.2_Gd_0.2_O_1.8_ is shown for understanding the polarization and oxide-ion transport nature of the sample (Fig. 6(c)). The linear tail present in the plot clearly suggests ionic conduction pathways. Thus the total conductivity in Gd and Bi co-doped ZrO_2_ sample is ionic in nature and facilitates oxide-ion conductivity due to oxygen vacancy migration. Further, we have also characterized the Zr_0.6_Bi_0.2_Gd_0.2_O_1.8_ sample heated in 10% hydrogen balanced in nitrogen atmosphere at 800 °C for 6 h by powder XRD study and we have not found any diffraction peaks for Bi metal in the XRD of the sample as all the peaks were identified to cubic phase of zirconia only (Fig. 6(e)). Further no colour changes were observed for the sample heated in H_2_ atmosphere at 800 °C for 6 h. These study clearly suggest the stability of the material in reducing media and also suggest that the total conductivity of our samples are predominantly an oxide-ion conduction as Gd^3+^ and Bi^3+^ doped sample contain oxide-ion vacancy. A comparison of oxide-ion conductivity of Zr_0.6_Bi_0.2_Gd_0.2_O_1.8_ in the same temperature range of 300–650 °C with other established oxide-ion conductors having crystal structures of fluorite or perovskite also presented in (Fig. 6(d)). Oxide-ion conductivity of Zr_0.6_Bi_0.2_Gd_0.2_O_1.8_ (ZBGO) is very much comparable to Sr and Ga doped LaGaO_3_; LSGM and KTa_0.4_Ti_0.3_Ge_0.3_O_2.7_ (KTTGO). The activation energy for oxide-ion conductivity was found as low as 0.42 eV. In the case of all samples, a sudden increase in oxide-ion conductivity was found at or around 450 °C.

**Fig. 7:**
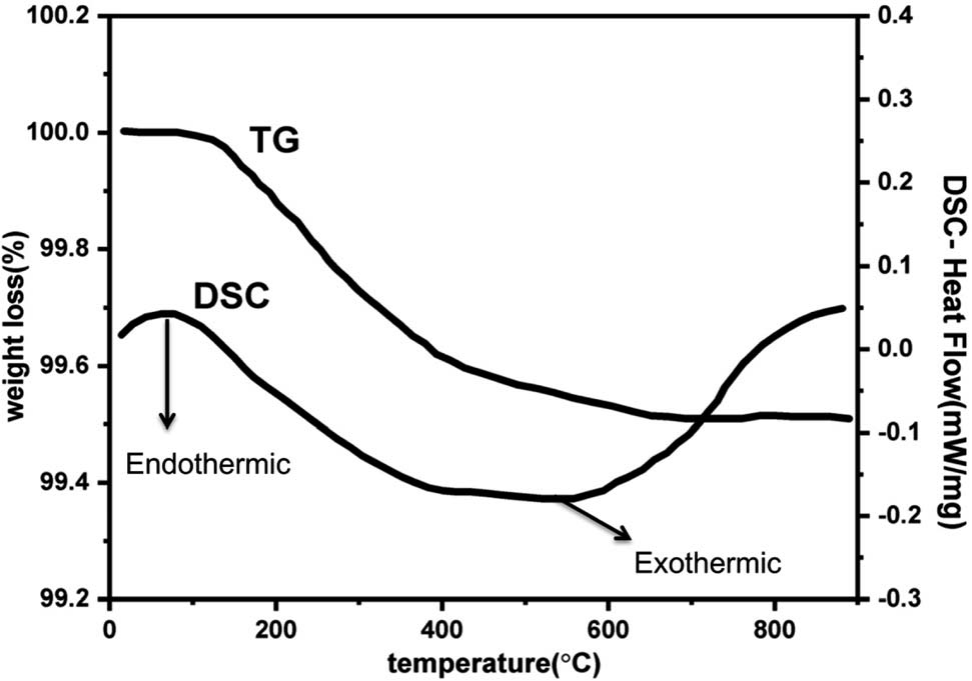
TGA and DSC curves of Zr_0.6_Bi_0.2_Gd_0.2_O_1.8_ preheated at 120 °C.

**Fig. 8:**
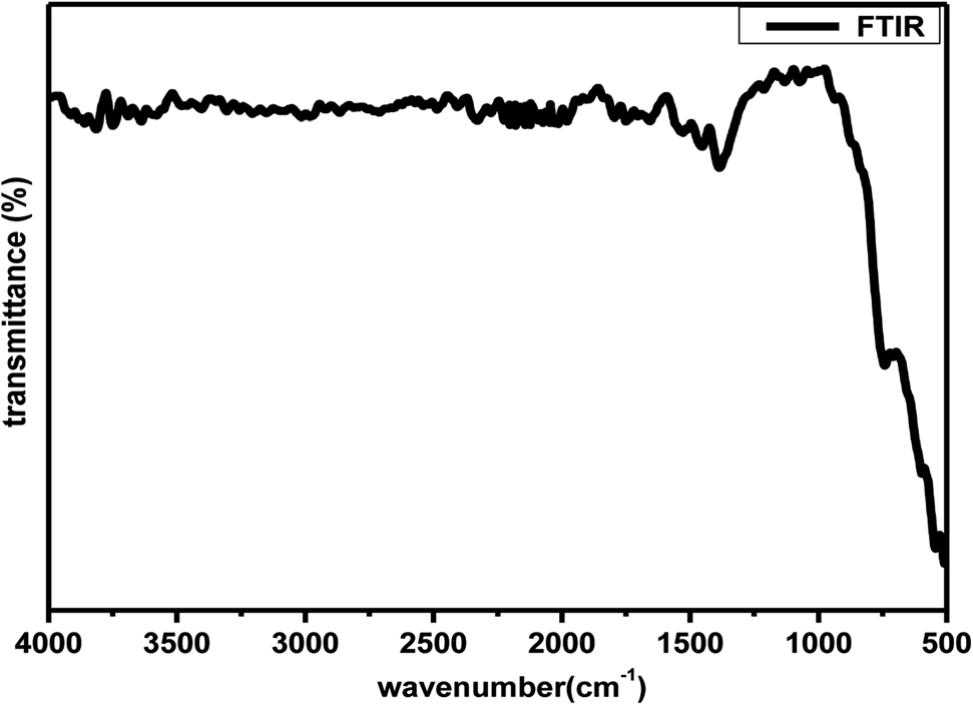
FT-IR spectra of Zr_0.6_Bi_0.2_Gd_0.2_O_1.8_ preheated at 120 °C.

Further to understand the sudden increase in conductivity if it is associated with any phase transformation, thermogravimetric and differential scanning calorimetry (TGA-DSC) analysis at a constant heating rate of 10 °C per minute in the temperature range of 30–900 °C in N_2_ atmosphere. Fig. 7 shows the TGA plot for Zr_0.6_Bi_0.2_Gd_0.2_O_1.8_ sample preheated at 120 °C. The lack of physically adsorbed water on the sample was demonstrated as marginal weight loss was observed up to 150 °C followed by very little weight loss (∼0.5%) up to 900 °C. The TGA analysis confirms the relatively low hygroscopicity or dry nature of the material. The DSC curve shown in Fig. 7 does not show any significant feature for any associated phase change that may arrive from oxide-ion vacancy or structure reorientation. Thus the TGA/DSC studies confirm the structure stability of the material in the temperature range of 30–900 °C. In addition, an FT-IR study was also performed to monitor the presence of hydroxide ions or water absorption at the oxygen vacancy position or at the surface of the Bi and Gd co-substituted ZrO_2_ samples. Fig. 8 displays the FT-IR spectra of Zr_0.6_Bi_0.2_Gd_0.2_O_1.8_ sample preheated at 120 °C for about 1 hour. Absence of peaks between 3300 and 4000 cm^−1^ clearly suggests the absence or insignificant presence of hydroxide ions or physio-adsorbed water on the surface of the material. This confirms that the conductivity observed for cubic fluorite Zr_1−*x*−*y*_Bi_*x*_Gd_*y*_O_2−(*x*+*y*)/2_ samples are only due migration of oxide-ion vacancies in the lattice.

**Fig. 9:**
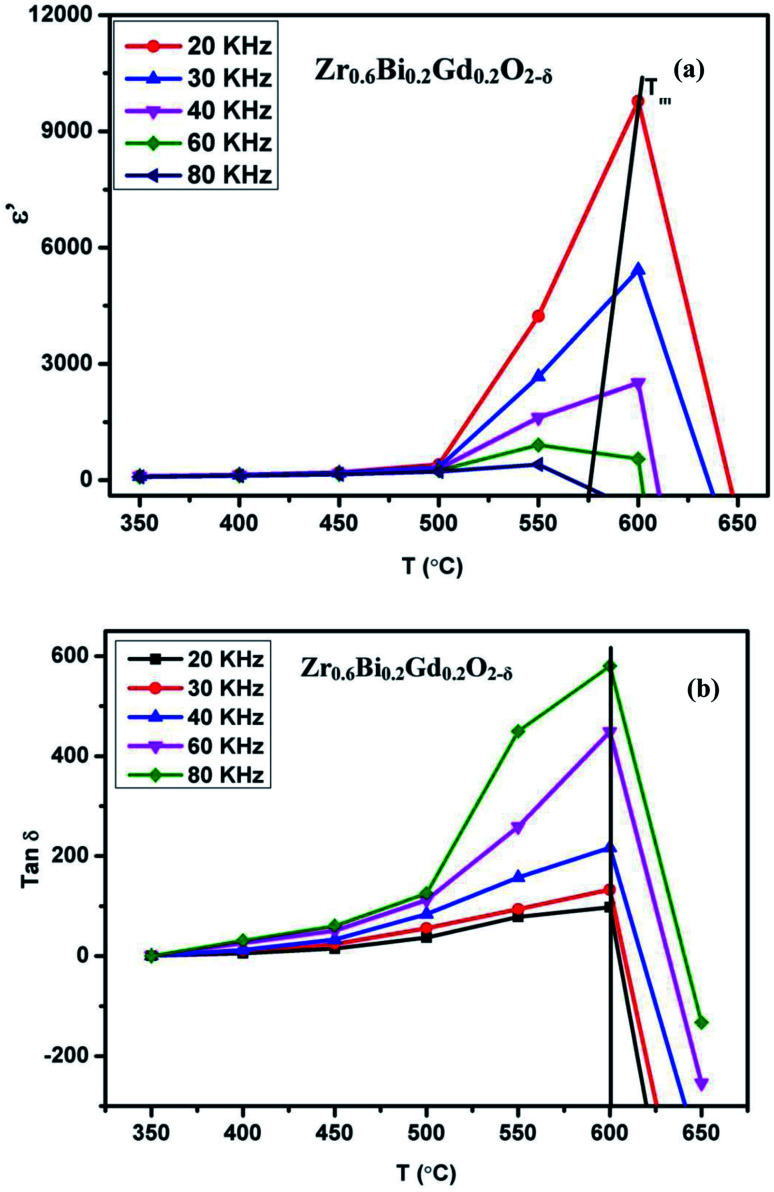
(a) Dielectric constant of Zr_0.6_Bi_0.2_Gd_0.2_O_1.8_ at different temperature and frequencies and (b) dielectric loss of Zr_0.6_Bi_0.2_Gd_0.2_O_1.8_ at different temperature and frequencies.

The sudden change or activation of oxygen vacancy migration above 450 °C confirms the oxide-ion transport within the percolation limit of a conductive phase transition coupled with thermal activation. Further to understand the effect of dielectric polarizability on oxide-ion conductivity, the dielectric constant in the frequency range of 20 kHz to 100 kHz at different temperatures is plotted in Fig. 9(a). The dielectric studies show a relaxor type behavior coupled with diffusive phase transition characterized by the permittivity dependence on the temperature and on applied frequencies. Zr_0.6_Bi_0.2_Gd_0.2_O_1.8_ sample show a significant 
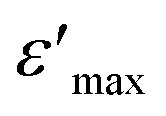
 (maximum permittivity) equivalent to ∼9725 at 600 °C at applied frequency 20 kHz, much higher than those for the pure ZrO_2_. With increasing applied frequency, the *T*_m_ had varied and observed to decrease. This gradual decrease with increasing applied frequency confirms the relaxor behaviour of this high κ dielectric material. Also, these compositions show a rather high dielectric loss (tan *δ* > 100) above ∼400 °C that increases exponentially with temperature above 600 °C (Fig. 9(b)) suggesting high leakage current at elevated temperatures. The dielectric relaxation of the dipole moment can lead to the material's superior oxide-ion conductivity at the temperature close to *T*_m_. The relaxation of net dipole moment generated over oxygen vacant octahedra can play a vital role in reorientation of the polyhedra at elevated temperature to provide the short transport pathways for the oxide-ion vacancy migration. Thus this giant loss (high leakage) seems to be associated with conduction or migration of oxide vacancy.

It is well known that higher concentration of oxide-ion vacancies at lattice sites and their high mobility are two key factors for achieving high ionic conductivity in typical oxide ion conductors. For cubic zirconium oxides, oxide ionic conduction is primarily associated with the conducting passageway through a cubic block and the movement of oxygen vacancies dominates. Similar to PZT-based ferroelectric ceramics, the vibration of a smaller-sized Ti^4+^ and Zr^4+^ cation from its mean position in octahedral coordination was shown to have the high dielectric constant and relaxor type behaviour and associated high oxide-ion conductivity in KTa_0.4_Ti_0.3_Ge_0.3_O_2.7_.^5^ Here, Bi^3+^ and Gd^3+^ ions were doped into ZrO_2_ lattice to stabilize the materials in cubic phase and the lone pair of Bi^3+^ ions can also play a vital role in enhancing the polarizability of the solid solution. Further, the synergistic interaction by introducing a secondary substituent (Gd^3+^ ions) seem to play important role to enhance the oxide-ion vacancy transport within the percolation limit ion transport inside the host structure at lower temperatures. High polarizability of Bi^3+^ with 6s^2^ lone pair electrons has been viewed as a key factor for high ionic conductivity in Bi-based oxide conductors, *e.g.*, δ-Bi_2_O_3_,^30^ Bi_4_Ti_3_O_12_,^5^ γ-Bi_4_V_2_O_11_.^47^ Similarly, in the present material, the 6s^2^ lone pairs of Bi^3+^ ions can be oppositely pointed toward a vacant central of the Zr plane of ZrO_8_ polyhedra in the parent-phase as shown in Fig. 4. Also, this structure can have a relaxed unit cell with longer Zr–O bonds, where the oxygen vacancy can jump by thermal activation to the energetically equivalent neighbouring oxygen sites of the lattice. However, more advanced structural and phase transition studies such as neutron powder diffraction (NPD) or EXAFS studies at various temperatures are important for the making mechanistic propositions about the associated phase transition responsible for sudden increase in the conductivity of the materials at elevated temperatures. However, the direct correlation of dielectric relaxation of dipole moments to superior oxide-ion transport was also observed previously for Na_0.5_Bi_0.5_TiO_3_,^1–3,27,28^ KTa_1−*x*−*y*_Ti_*x*_Ge_*y*_O_3−*δ*_,^5^ 20% Sm doped CeO_2_(Ce_0.8_Sm_0.2_O_2−*δ*_)^6^ and La_2_Mo_2_O_9_.^7^ However further studies are necessary to validate the relationship of dielectric relaxation and associated phase transitions that provide shorter conduction pathways for material's superior oxide-ion conductivity.

## Conclusions

Cubic Zr_0.6_Bi_0.2_Gd_0.2_O_1.8_ was synthesized by solution combustion synthesis route and characterized as high κ relaxor dielectric as well as an oxide-ion conductor for IT-SOFCs applications using various characterization techniques such as powder XRD, SEM-EDX and impedance spectroscopy. Only up to 40% ions can be substituted at Zr site to make single phase cubic fluorite material using Gd^3+^ and Bi^3+^ as simultaneous substituent. Up to 20% of Bi and 30% Gd was co-substituted in ZrO_2_ lattice (Zr_1−*x*−*y*_Gd_*x*_Bi_*y*_O_2−*δ*_, *x* + *y* ≤ 0.4, *x* ≤ 0.3 and *y* ≤ 0.2) in different combinations and several solid solutions were synthesized in cubic fluorite structure. Zr_0.6_Bi_0.2_Gd_0.2_O_1.8_ sample showed superior oxide-ion conductivity with the lower activation energy in the temperature range of 300–650 °C. The oxide-ion conductivity of Zr_0.6_Bi_0.2_Gd_0.2_O_1.8_ was found 10^−2^ S cm^−1^ at or above 500 °C. Considering the robustness of ZrO_2_ based systems, the materials can act as an possible candidates as an oxide-ion electrolyte for intermediate temperature solid oxide fuel cells (IT-SOFCs), as the material requires low processing cost and delivers high conductivity at relatively lower temperatures. Nonetheless, more studies are required to determine the applicability of the materials as an oxide-ion electrolyte for the production of IT-SOFCs. The cubic Zr_1−*x*−*y*_Bi_*x*_Gd_*y*_O_2−*δ*_ phase also showed relaxor type high κ dielectric behaviour (*ε*′ = 9725 at 600 °C at applied frequency 20 kHz for Zr_0.6_Bi_0.2_Gd_0.2_O_1.8_) with *T*_m_ approaching to 600 °C. The polarizability of Bi^3+^ ion coupled with high κ dielectric relaxation (high dielectric leakage) can utilize as new tool to develop superior oxide-ion conduction near *T*_m_ (the temperature of the maximum dielectric permittivity) and effort can be made to bring down the *T*_m_ to achieve higher ionic conductivity at lower temperatures.

## Conflicts of interest

The authors declare that they have no known competing financial interests or personal relationships that could have appeared to influence the work reported in this paper.

## Supplementary Material
